# TV or not TV? health information, anxiety and stress during the initial stage of COVID-19 epidemic in Italy

**DOI:** 10.1192/j.eurpsy.2021.727

**Published:** 2021-08-13

**Authors:** I. Piretti, F. Ambrosini, R. Sant’Angelo

**Affiliations:** 1 Psychologist, Hr Specialist, Independent Researcher, Cesena, Italy; 2 Psychologist, Independent Researcher, Savignano, Italy; 3 Mental Health Department, AUSL ROMAGNA, Cesena, Italy

**Keywords:** health information, TV, Internet, emotional contagion

## Abstract

**Introduction:**

With the spread of the coronavirus pandemic, there has been the dissemination of an enormous amount of information, through multiple channels, from different sources and with an often unverifiable basis (infodemic). In recent years, there has been debate in the literature about the possibility that different information channels (social media vs traditional media) can determine a more or less extensive emotional contagion regardless of the severity and direct exposure to the stressful event and more precisely through a ‘secondhand’ exposure to events.

**Objectives:**

We want to investigate whether the information channel or the amount of time dedicated to the update is associated with greater psychological sequelae.

**Methods:**

This study is based on a cross-sectional online survey conducted anonymously in the period between the tenth and seventeenth day of shelter in place in Italy. We used Zung Anxiety Self-Assessment Scale and Perceived Stress Scale 4. SPSS 21.0 was used for data analysis.

**Results:**

We collected data on 1047 individuals. In our sample, the Internet was the primary health information channel (55%) followed by TV (36%). Most TV and internet users spend about 1-3 hours a day for the update. There was no correlation between the time spent or the information channel used and higher levels of stress and anxiety.

**Conclusions:**

In our opinion, this relationship between the time spent receiving information or the most widely used information channel and psychological disorders is not clear. Is it the news channel causing an emotional contagion or are the most anxious people looking for news anywhere and anytime?

